# Artificial Intelligence and the Global Radiotherapy Gap

**DOI:** 10.1200/GO-26-00181

**Published:** 2026-07-09

**Authors:** Michael Benedict A. Mejia, Luisa E. Jacomina, Milit S. Patel, Fabio Y. Moraes, Sharif Elguindi, Nancy Y. Lee, Edward Christopher Dee

**Affiliations:** ^1^ Department of Radiation Oncology, Benavides Cancer Institute, University of Santo Tomas Hospital, Manila, Philippines; ^2^ Department of Molecular Biosciences, The University of Texas at Austin, Austin, TX; ^3^ Department of Radiation Oncology, Memorial Sloan Kettering Cancer Center, New York, NY; ^4^ Department of Radiology and Oncology, University of Sao Paulo, São Paulo, Brazil; ^5^ Department of Oncology, Queen's University, Kingston, ON, Canada; ^6^ Department of Medical Physics, Memorial Sloan Kettering Cancer Center, New York, NY

Radiotherapy is a clinically essential and cost-effective cornerstone in addressing the global cancer burden.^1-3^ This cost-effectiveness may be especially important in resource-limited settings, where patients with cancer and broader health systems face myriad challenges.^4,5^ Despite this, access to radiotherapy in developing economies has consistently been shown to be inadequate.^5,6^ Shortages in the number of megavoltage machines, brachytherapy units, radiation oncologists, medical physicists, dosimetrists, oncology nurses, and radiation therapists represent challenges in addressing the growing cancer burden.^5,7^ These shortages are even more pronounced in developing economies where both radiotherapy units and workforce shortfalls are greatly magnified.^6-9^

Strategies to improve access to radiotherapy are key, in the broader context of health system strengthening.^10-12^ Using artificial intelligence (AI) to automate processes that otherwise require manual intervention can help increase throughput and lessen the strain on already stretched human resources.^13^ The Radiation Planning Assistant (RPA) represents one concrete effort to translate this vision into practice.

In *JCO Global Oncology*, Netherton et al^14^ present an end-to-end, AI-enabled RPA that automates contouring, treatment planning, and quality assurance for cervical and prostate cancers, with the explicit goal of improving radiotherapy efficiency, consistency, and access in resource-limited settings.^14,15^ The RPA represents an integrated automation pipeline rather than a single algorithm, combining machine learning–based segmentation with automated treatment planning and workflow processes. In retrospective validation, most autocontours and plans were clinically acceptable with no or only minor edits, and the platform reduced the planning process from what can take days to weeks down to <2 hours, suggesting meaningful potential to reduce treatment planning burden and streamline workflows where workforce shortages are severe. At the same time, the findings support cautious implementation because expert review remains essential, certain structures and clinical scenarios remain challenging, and real-world evaluation will be critical before widespread adoption.^14,15^

The study's team has already demonstrated in previous publications how automation can potentially make radiotherapy workflows more efficient in real-world settings.^15^ In the present study,^14^ the authors introduce a more robust iteration of the program focusing on cervical and prostate malignancies using deep learning–based segmentation (nnU-Net) and knowledge-based planning, aiming to create automatically generated and globally acceptable target and organ-at-risk contours along with deliverable plans at a fraction of the time usually required. This approach could translate to more streamlined workflows and help scale up access and relieve an overworked planning and radiation oncology workforce in resource-constrained settings.

Building on the RPA's important work, reliable automation also has the potential to redirect manpower efforts toward ensuring the quality delivery of autogenerated plans. In the current era of precision radiotherapy, the delivery of intensity-modulated radiation therapy with ever-tighter margins requires constant vigilance to ensure setup reproducibility and regular verification. Reducing the burden on contouring, plan generation, and plan approval may allow efforts to shift toward treatment verification and on-treatment quality assurance. For instance, in pelvic radiotherapy, bladder filling and rectal emptying are important contributors to daily set up variability, and these factors should be considered in future efforts toward automation. Such advances could constitute technological task shifting.

There is, however, a need to tread with caution in the widespread application of automation and the adoption of AI, especially in resource-strained settings where opportunities for human oversight may be limited by manpower and subspecialty expertise.^6,13,16^ While the contours and plans generated by the models may be deemed acceptable by established guidelines (EMBRACE II, NRG-GU009, ESTRO ACROP, etc), the Dice similarity coefficient and 95% Hausdorff distance values remain below ideal ranges, indicating variability between autogenerated and manual contours and therefore necessitating edits.^14^

Notably, any edits were detected and made by gynecologic and urologic radiation oncology subspecialists. Such edits may potentially escape the attention of generalists, particularly in strained global community settings, despite the possibility that some of these discrepancies could affect outcomes. The limitations of the RPA have been appropriately described in the study.^14^ These limitations, however, may be particularly salient in settings where advanced disease is the norm and gross nodal disease—especially among patients with cervical cancer—is rather common.

Importantly, while the RPA's web-based design reduces the hardware and software burden on participating centers, automation alone cannot overcome shortages in treatment machines, imaging, maintenance systems, and broader radiotherapy infrastructure.^6^ Several studies highlight the many and heterogenous challenges faced by developing cancer systems, including adequacy of facilities; availability of trained physicists, dosimetrists, therapists, nurses, and radiation oncologists; and opportunities for peer review, quality assurance, and the adoption of modern treatment concepts such as hypofractionation.^5,17,18^ The integration of newer technologies to address gaps in treatment delivery can help to compensate for shortfalls in the availability of human capital, but must be matched with efficient resource allocation and cancer system strengthening more broadly.^6^

The development of the RPA has been particularly thoughtful in that the team did not approach the problem solely through the lens of more resourced settings but instead engaged with the realities faced by patients and clinicians in many global contexts, specifically in South Africa.^14^ Earlier efforts also attempted to address similar situations in other environments.^19^ End-to-end involvement of stakeholders on the ground strengthens the validity of the effort by ensuring that the actual needs of the intended beneficiaries are met.^20^ Future work in this domain should continue to emphasize data set representativeness, to minimize potential algorithmic bias, and to reinforce the need for clear clinician oversight.

While the introduction of robust AI and automation innovations may allow better throughput and more consistent generation of contours and plans, the capacity to perform critical human intervention necessary for delivering high-quality radiotherapy at scale must also be developed and optimized. A cautious and gradual rollout of such innovative strategies, with clearly identified evaluation points, may be the most prudent approach before wider application.

Importantly, broader deployment will require not only external validation across different clinical environments, including practice settings, patient populations, and radiotherapy infrastructure contexts, but also imaging protocols, disease stage distribution, and patterns of nodal spread.^20-23^ Implementation of RPA and parallel efforts will benefit from critical, ideally prospective, evaluation in routine practice. Prospective implementation studies evaluating clinical outcomes, treatment delays, workflow efficiency, and user acceptance will be essential to determine whether automated planning systems translate into meaningful improvements in real-world cancer care.

In the future, the eventual development of linear accelerator-end AI systems—such as automated matching and adjustment as well as broader adoption of adaptive planning^24^—may further enhance these efforts toward more streamlined workflows that may facilitate the delivery of quality radiotherapy at scale.^25,26^

An idea is only as valuable as its execution. The RPA is a promising step in the right direction, with development marked by global partnerships and careful evaluation. Ultimately, future efforts must also be directed toward ensuring applicability, realistic implementation, sustainability of gains, and continuous evaluation to continue to address remaining gaps in global access to radiotherapy.
